# Decellularized Splenic Matrix as a Scaffold for Spleen Bioengineering

**DOI:** 10.3389/fbioe.2020.573461

**Published:** 2020-10-02

**Authors:** Tadeu Ériton Caliman Zanardo, Fernanda Gobbi Amorim, Gabriel Henrique Taufner, Rayssa Helena Arruda Pereira, Ian Manhoni Baiense, Afrânio Côgo Destefani, Leo Kei Iwai, Raul Cavalcante Maranhão, Breno Valentim Nogueira

**Affiliations:** ^1^Biotechnology Graduate Program, Rede Nordeste de Biotecnologia (RENORBIO), Vitória, Brazil; ^2^Tissue Engineering Core, Department of Morphology, Federal University of Espírito Santo, Vitória, Brazil; ^3^Pharmaceutical Sciences Graduate Program, University of Vila Velha, Vila Velha, Brazil; ^4^Laboratory of Proteomics and Mass Spectrometry-Special Laboratory of Applied Toxinology LETA/CETICS, Instituto Butantan, São Paulo, Brazil; ^5^Heart Institute (InCor), Medical School Hospital, University of São Paulo, São Paulo, Brazil

**Keywords:** splenic scaffold, decellularization, extracellular matrix, proteomic analysis, recellularization

## Abstract

The spleen is considered a non-essential organ. However, its importance is increasingly clear, given the serious disorders caused by its absence or dysfunction, e.g., greater susceptibility to infections, thromboembolism and cancer. Surgical techniques to preserve the spleen and maintain splenic function have become increasingly common. However, the morbidity and mortality associated with its absence and dysfunction are still high. We used the decellularization technique to obtain a viable splenic scaffold for recellularization *in vitro* and propose the idea of bioengineered spleen transplantation to the host. We observed the maintenance of important structural components such as white pulp, marginal zone and red pulp, in addition to the network of vascular ducts. The decellularized scaffold presents minimal residual DNA and SDS, which are essential to prevent immunogenic responses and transplantation failure. Also, the main components of the splenic matrix were preserved after decellularization, with retention of approximately 72% in the matrisomal protein content. The scaffold we developed was partially recellularized with stromal cells from the spleen of neonatal rats, demonstrating adhesion, proliferation and viability of cells. Therefore, the splenic scaffold is very promising for use in studies on spleen reconstruction and transplantation, with the aim of complete recovery of splenic function.

## Introduction

The spleen is a lymphoid organ known to play a role in erythrocyte homeostasis and iron metabolism. It also acts as a filter and in the generation of antigen-specific immune responses that protect the body against blood-borne bacterial, viral and fungal infections (Bronte and Pittet, 2013). Recently, its collapse has been demonstrated in cases of Coronavirus Disease 2019 (COVID-19) and experimental model of SARS-CoV-2 infection, where splenic atrophy has been observed, as well as clinical lymphopenia (Chan et al., 2020; Guan et al., 2020; Xu et al., 2020).

Total absence of spleen, usually by splenectomy, and hyposplenia, are associated with several diseases, such as sickle cell anemia, cancer and HIV infection (Kyaw et al., 2006; Thomsen, 2009; Di Sabatino et al., 2011). Deficiency of splenic function may predispose to thromboembolic events, and often leads to immunological deficiencies (Di Sabatino et al., 2011). Thus, infections caused by encapsulated bacteria refractory to antibiotical therapy prone to occur and may near high morbimortality rates between 40 and 54% (Di Sabatino et al., 2011; Taniguchi et al., 2014). Knowing the crucial role of the spleen in the immune response, splenectomy also becomes a risk factor for some types of cancer (Mellemkjaer et al., 1995; Kristinsson et al., 2014; Sun et al., 2015). In this setting, surgical techniques to preserve the spleen are becoming increasingly common. In cases of splenectomy caused by trauma, surgeons should attempt to save as much tissue as possible, always resorting to splenosis where feasible (Di Sabatino et al., 2011). However, splenosis appears to offer little protection against post-splenectomy infection (Connell et al., 2011).

Regenerative medicine rises an excellent alternative for regenerating, repairing or replacing diseased or absent organs and tissues (Mao and Mooney, 2015; Aamodt and Grainger, 2016; Garreta et al., 2017). It is known that the decellularized extracellular matrix (ECM) can modulate cellular behavior, stimulating cell binding, migration, proliferation, and differentiation (Hussey et al., 2018; Heath, 2019). ECM is considered the “backbone” of the spleen, playing an important role in immune compartmentalization, but little is known about its constitution (Lokmic et al., 2008; Song et al., 2013).

A biomaterial from ECM can be produced through organ decellularization, generating a three-dimensional scaffold, with the removal of much of the DNA and cell content. This process preserves the structure of vascular ducts and important molecules such as collagens, glycoproteins, and proteoglycans (Ott et al., 2008; Hillebrandt et al., 2019). The decellularized scaffold can then be recellularized with the patient own cells, thereby preventing rejection (Gilpin and Yang, 2017; Hussey et al., 2018). Thus, reconstruction of the spleen from decellularized scaffolds can solve the problem of splenosis caused by the lack of sufficient amount of tissue to ensure protection against infections.

Despite of the physiological importance of the spleen, studies aimed to reconstitute the organ are lacking in the literature. This prompted us to use a decellularization technique to create a viable splenic scaffold that, in a pioneering way, can enable spleen reconstruction with the cells from the host itself.

## Materials and Methods

### Animals

All animal experiments were carried out following the guidelines for biomedical research of the Brazilian Society for Experimental Biology and approved by the Institutional Ethics Committee of Universidade Federal do Espírito Santo (CEUA-UFES no. 042/2016). Healthy male Wistar rats, aged 8–10 weeks, weighing between 250 and 350 g, were used for the spleen removal and decellularization studies (*n* = 34). For the isolation of splenic stromal cells, neonatal Wistar rats (3 days old) were sacrificed by decapitation (*n* = 6).

### Decellularization

**FIGURE 1:**
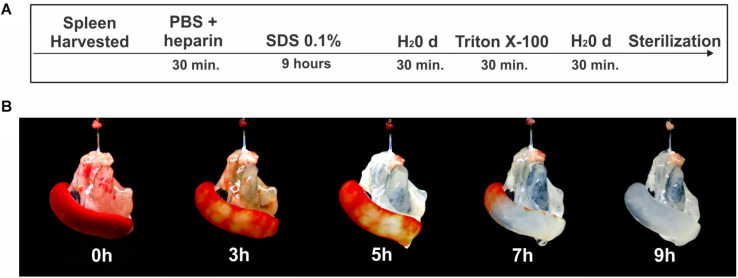
Process of decellularization of the spleen. **(A)** Schematic representation of the protocol used for splenic decellularization. **(B)** Representative images of the splenic decellularization process over time.

For the spleen decellularization, the animals were euthanized with an intraperitoneal injection of anesthetic ketamine (100 mg/kg) and xylazine (10 mg/kg), followed by 1,000 units of heparin i.p. to prevent clot formation. Subsequently, under aseptic conditions, a laparotomy was performed followed by cannulation of the splenic artery with a cannula (Micro-Renathane – 0.040 mm OD × 0.025 mm ID; Braintree). After removal, the spleen was perfused with a solution of phosphate buffered saline (PBS) with heparin (100 Units/mL) for 30 min with the aid of a peristaltic pump (Gilson^®^) at a rate of 1.2 mL/min. Subsequently, the organ was perfused with 0.1% SDS detergent (sodium dodecyl sulfate) for 9 h and triton X-100 1% for 30 min, always diluted in distilled water (H_2_Od). H_2_Od was infused for 30 min between the detergent perfusion steps (Figure 1A). For the final washing of the scaffold, sterile PBS pH 7.4 was perfused for 60 min. The infusion pressure of all detergents and solutions in the organ was about 100 mmHg (± 10 mmHg), which was verified throughout the processing with a manometer connected to the system. The flow was monitored and maintained at approximately 1.5 mL/min.

### Histological Analysis

Samples of native, decellularized and recellularized tissue were fixed and incorporated into Historesin (Leica, Germany). After polymerization, 3 μm thick sections were removed from each tissue using a microtome (Leica RM 2125, Germany). The used stains were hematoxylin and eosin, Picrosirius red and periodic acid-Schiff (PAS). For immunohistochemistry, the following protocol was followed: toluene; xylene, and acetone (PA, for 30 min); rehydration in decreasing series of ethanol (96, 70, 50, and 30%) and distilled water. Subsequently, antigenic recovery was performed with citrate buffer pH 6.0. The endogenous peroxidase (hydrogen peroxide block, Catalog n° DHP-125, Spring Bioscience, Canada) and non-specific proteins were blocked (Protein Block, Catalog n° DPB-125, Spring Bioscience, Canada) and the slide samples were incubated with primary antibodies anti-fibronectin and anti-laminin (Abcam, Cambridge, United Kingdom), both diluted 1:100 with Antibody Diluent (Catalog n° ADS-125, Spring Bioscience, Canada) overnight at 4°C. Subsequently, the samples were incubated with the secondary antibody (N-Histofine polymer – Nichirei Biosciences Inc., Japan), revealed with the substrate and contrasted with hematoxylin. All images were obtained using an optical microscope (AX70, Olympus, Japan) coupled to a video camera (AxioCam ERc5s, Carl Zeiss, Germany).

### Determination of Residual DNA

The DNA content in native (*n* = 4) and decellularized (*n* = 6) tissues was extracted using the standardized saline extraction method (Bruford et al., 1992). Briefly, the tissues were lyophilized, cut into small fragments and isolated with extraction buffer (1 M tris, pH = 8; 5 M NaCl; 0.5 M EDTA, pH = 8) and lysis buffer (extraction buffer, 10% SDS and proteinase K). After digesting the tissue overnight, the DNA was precipitated with 5 M NaCl; the precipitated protein content was discarded, and the remaining DNA was diluted in 100 μL of distilled water. The total amount of DNA was quantified using the nucleic acid detection function in the NanoDrop 2000 spectrophotometer (Thermo Scientific, United States).

### Determination of Residual SDS Content

To determine the residual SDS concentration in the scaffold (*n* = 3), the methylene blue (MB) method was used (Zvarova et al., 2016). About 2 mL of the organ effluent was collected at two different times during the decellularization process: 1) Last 1 mL of 0.1% SDS used during the decellularization process; 2) Last 1 mL of distilled water used in the final wash of the scaffold. For the standard curve, the SDS was diluted in deionized water in series, starting from a 1% SDS solution up to its thousandth part (∼0.001%). Washing aliquots (in triplicate) were then mixed with a solution containing 0.0125% MB (Sigma-Aldrich) in deionized water (v/v) in a ratio of 1:100 (5 μL of effluent + 495 μL of MB). After stirring, chloroform was added in a 1:2 ratio (1 mL of chloroform) and incubated for 30 min at room temperature. After incubation, 150 μL of the lower layer (chloroform) was extracted, placed in a 96-well plate and read spectrophotometrically in a microplate reader (Varioskam Flash, Thermo Fisher) at 650 nm.

### Collagen Quantification

The amount of collagen was verified indirectly by determining the content of the amino acid 4-hydroxyproline present in the tissues (Samuel, 2009; Carlson, 2014). In the standard curve, dilutions were made from a solution containing 2,600 μg/mL of hydroxyproline to a concentration of 2.5 μg/mL. Subsequently, the native and decellularized tissues (*n* = 4) were previously lyophilized and about 10 mg of each sample (in triplicate), in addition to the blank and the standards, was incubated in 50 μL of 7 N NaOH and autoclaved at 120°C (*p* ≈ 98.1 kPa) for 40 min for alkaline hydrolysis. Subsequently, about 50 μL of 3.5 M H_2_SO_4_ was added to the tubes to acidify the hydrolyzate. Hydroxyproline was oxidized to pyrrole after adding 450 μL of chloramine T 0.0025 M in citrate-acetate buffer pH 6.0 for 25 min at room temperature. 500 μL of 0.5 M Ehrlich reagent (4-dimethylaminobenzaldehyde in perchloric acid/2-propanol) was added to the tubes and incubated in a water bath at 60°C for 15 min, forming a red-purple compound. The absorbance of the samples was read immediately, spectrophotometrically, on a microplate reader (Varioskam Flash, Thermo Fisher) at 560 nm.

### Quantification of Glycosaminoglycans

The quantification of GAGs was performed by incubating the samples in Alcian Blue with subsequent spectrophotometric reading according (Karlsson and Björnsson, 2001). For the standard curve, serial dilutions were performed from a chondroitin-6-sulfate solution at a concentration of 0.4 to 0.0125 mg/mL. About 5 mg of lyophilized samples of native and decellularized tissues (*n* = 4) (in duplicate) was precipitated in guanidine-HCl buffer 8 M pH 1.5 for 24 h under agitation. After centrifugation, the precipitate was incubated with a solution of Alcian Blue (8GX, Sigma-Aldrich, United States) and stored overnight at 4°C. About 5.0 mL of Alcian blue stock solution (Alcian blue 2% in 0.1% H_2_SO_4_/0.4 M guanidine-HCl) was used to prepare the Alcian blue reagent (Alcian blue in 0.1% H_2_SO_4_/0.25% Triton), which was later incubated with the sample. Excess stain was removed by washing in DMSO. After dissociating the GAGs-Alcian Blue complexes in the 4 M guanidine HCl/propanol solution, about 240 μL of the tissue samples, standard and blank (water), were placed in a 96-well plate and read spectrophotometrically in a microplate reader (Varioskam Flash, Thermo Fisher) at 605 nm. The amount of GAGs present in the samples was directly proportional to the intensity of Alcian Blue linked to the GAGs.

### Ultrastructural Analysis

For transmission electron microscopy (TEM), samples of native and decellularized tissue were collected and fixed in Karnovsky’s solution (2.5% glutaraldehyde, 2% paraformaldehyde, and 0.1 M cacodylate buffer). Subsequently, the tissues were post-fixed in osmium tetroxide, dehydrated in ethanol (30, 50, 70, 90, and 100% – 30 min each) and soaked in epoxy resin (EMbed 812, Electron Microscopy Sciences). Ultrafine sections (60 nm thick) were obtained on an ultramicrotome (UCT, Leica Microsystems). Next, uranyl acetate (5%, pH 5 – 30 min) and lead citrate (2%, pH 12 – 5 min) contrast were added. Electronic micrographs representative of the different groups was obtained using a transmission electron microscope (JEOL JEM-1400, Japan) at 80 kV.

For scanning electron microscopy (SEM), after the fixation steps, the cryoprotectant (30% glycerol and 0.1 M cacodylate buffer) was added and the samples were placed in a freezer at −80°C for total tissue freezing. For sample fracture, liquid nitrogen was poured directly onto the tissue. This was followed by dehydration in ethanol (30, 50, 70, 90, and 100% – 30 min each), drying at the critical point of CO_2_ (Autosandri-815, Tousimis), coating with 10 nm of pure gold in a sprinkler at vacuum (Desk V, Denton Vacuum) and analysis in direct mode using a scanning electron microscope (Jeol, JEM-6610 LV). To determine the diameter of the decellularized tissue matrix fibers (*n* = 3), the Diameter J plugin of the Image J software (National Institutes of Health, Bethesda, MD, United States) was used. For the analyses, 45 images were obtained randomly from the samples in an increase of 10,000×.

### Proteomic Analysis

For proteomic analysis, lyophilized samples of native and decellularized tissues (*n* = 4) were processed according to the modified protocol of Barallobre-Barreiro et al. (2017). First, cellular contaminants in native tissues were removed. Briefly, about 50 mg of each native tissue was washed with 0.5 M NaCl in 10 mM Tris pH 7.5 (1:10 p:v) and incubated on a shaker for 4 h. About 1 mL of 0.1% SDS with 100 mL of 25 mM EDTA was added to the samples and incubated on the shaker at 500 rpm for 16 h (overnight). After centrifuging and discarding the supernatant, the native tissue was then washed with 1 mL of deionized H_2_O to remove the SDS. Subsequently, about 500 μL of 4 M guanidine hydrochloride (GnHCl) with 50 mM sodium acetate pH 5.8 and 50 μL of 25 mM EDTA was added to the samples of native and decellularized tissue to extract the protein content from the matrix, after incubation for 48 h at room temperature in the vortex. Next, the protein quantification of the samples was performed in a Nanodrop 2000 spectrophotometer (Thermo Scientific, United States) at 280 nm with an extinction coefficient 1.1. The ethanol precipitation steps, denaturation with 9 M urea and 3 M thiourea, reduction with 100 mM dithiothreitol (DTT), alkylation with 0.5 M iodoacetamide and digestion with trypsin (0.01 μg/μL) were followed. The desalination step was performed with ZipTips C18 (Millipore, Billerica, MA, United States).

Shotgun proteomics was performed on the LTQ-Orbitrap Velos mass spectrometer coupled to EASY-nLC II liquid chromatography (ThermoFisher, Waltham, MA, United States). Data were collected in a data acquisition-dependent mode in which the five most intense precursor ions of each cycle were selected for fragmentation by collision-induced dissociation (ICD) for 90 s; a nanospray voltage of 2.3 kV was selected, at a temperature of the 250°C, to ionize the sample. The injection times in the trap ion and FT-MS were 100 ms each, with a resolution of 30,000 through the 300–1,800 m/z range.

The analysis of the raw data was performed in the software program Peaks Studio 8.5 using the following databases: 1) “*Rattus norvegicus*” in Uniprot (36,879 results in July 2018; 2) “extracellular matrix + *Rattus norvegicus*” in NCBI (836 results in July 2018; and 3) “proteoglycans + *rattus norvegicus*” at NCBI (76 results in July 2018). Carbamidomethylation was set as fixed modification, while oxidation (M) was set as variable modification. Maximum cleavages were selected in three for trypsin. The precursor mass error and the fragments were 10 ppm and 0.5 Da, respectively. A 1% false discovery rate (FDR) with single peptides ≥1 was used to filter low-quality proteins in the PEAKS mode analysis. All the proteomic raw data were deposited on jPOSTrepo repository^1^ under the accession numbers of JPST000915 and PXD020363 for ProteomeXchange.

### Cell Isolation

For cell isolation, 3-day-old neonatal rats were sacrificed by decapitation and dipped in 70% alcohol. They were then taken to a laminar flow cabinet where the peritoneum was exposed with the aid of a scalpel. Subsequently, the spleen was perforated in a petri dish and the fragments dipped in a buffer containing 0.45 mg/mL Liberase TL (Roche) and 0.5 mg/mL DNase I (Roche) in DMEM medium (Dulbecco’s Modified Eagle Medium, Sigma-Aldrich, United States). Digestion was performed under constant agitation (140 rpm) for 30 min at 37°C and complete disintegration was obtained by pipetting to obtain a single-cell suspension. The cells were cultured in a culture medium containing DMEM plus 20% fetal bovine serum (FBS) and 1% antibiotic/antimycotic (penicillin 10,000 Units, streptomycin 10 mg, and 25 μg amphotericin B/mL), with the removal of non-adherent cells and change of medium after 3–4 days. In all experiments, adherent cells (stromal cells) were used in the passages P1–P5.

### Recellularization

The first cell seeding method tested was perfusion (*n* = 3). To sterilize the scaffold, a 0.2% peracetic acid solution was perfused for 15 min, followed by bathing in ultraviolet light for another 15 min. For the final washing of the scaffold, sterile PBS pH 7.4 was perfused for 60 min. After, the scaffold was perfused with complete DMEM culture medium for 30 min. The cell perfusion process was performed in the cannulated splenic artery in stages, starting with three infusions of 1 mL of complete DMEM medium containing 1 × 10^6^ stromal cells/mL, with a sterile syringe, with 10-min intervals between each infusion, followed by recirculation of complete DMEM medium for 10 min with a flow rate of 3 mL/min. The infusion and recirculation protocol was performed twice, totaling six cellular infusions. After the infusions, the organ was immersed in complete DMEM medium and taken to the incubator at 37°C and 5% CO_2_ for 24 h. Next, the scaffold was fragmented into pieces of approximately 1 cm^3^ and grown and cultured at 37°C and 5% CO_2_, for 1 and 5 days.

The second method of cell seeding tested was the direct cultivation of stromal cells on the scaffold (*n* = 3). For this, the previously sterilized scaffold with approximately 1 cm^3^, which were seeded with 100 μL of DMEM containing 1 × 10^6^ cells. For complete cell adhesion, the scaffold was left for 2 h in the incubator at 37°C and 5% CO_2_, complete DMEM medium was added and cultured for 1 and 5 days.

### Alamar Blue Assay

The Alamar Blue assay (Invitrogen, Carlsbad, CA, United States) was used to monitor the viability and proliferation of stromal cells cultured along with the scaffold (*n* = 3). About 5 × 10^4^ stromal cells were seeded on fragments of scaffolds measuring approximately 1 cm^3^, in a 48-well plate (Kasvi) and then cultured at 37°C in a humidified atmosphere containing 5% CO_2_ in complete DMEM medium. After 1 and 5 days in culture, the medium was removed, and a new medium added with the Alamar Blue reagent (180 μL of complete DMEM medium and 20 μL of Alamar blue). After 4 h of incubation in the incubator at 37°C and 5% CO_2_, 200 μL of the solution from each well were transferred to a 96-well plate (Corning, Costar). The fluorescence intensity was measured using a microplate reader (Synergy H1 Hybrid Multidetection Reader – BioTek) with 560 nm of excitation and 590 nm of emission.

### Statistical Analysis

All data were expressed as mean ± standard error of the mean. Statistical analysis was performed using Student’s *t*-test for independent samples and one-way and two-way ANOVA followed by Bonferroni’s test for multiple comparisons. Statistical analyses were performed using the Prism software (Prism 6, GraphPad, San Diego, CA, United States). Differences were considered significant when *p* < 0.05.

## Results

**FIGURE 2:**
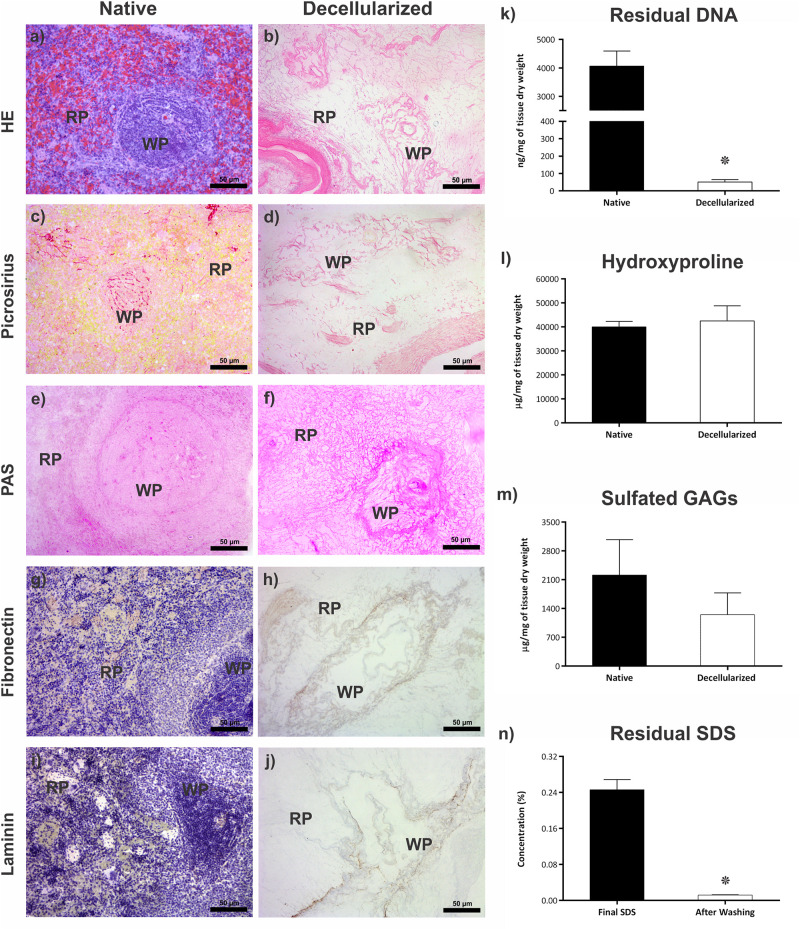
Evaluation of the decellularization process. **(a,b)** Representative images of the native spleen and decellularized scaffold stained by HE, showing the presence of cells with their nuclei in the native and their absence with the maintenance of ECM in the decellularized scaffold. **(c,d)** Representative images of the native organ and the scaffold stained with Picrosirius red, demonstrating the preservation of collagen fibers after decellularization. **(e,f)** Representative images of native organ and scaffold stained with PAS, showing the preservation of glycoproteins after decellularization. **(g,h)** Representative images of immunohistochemistry analysis of fibronectin glycoprotein in native tissue and its maintenance after decellularization. **(i,j)** Representative images of immunohistochemistry analysis of laminin glycoprotein in native tissue and its maintenance after decellularization. **(k)** Bar graph showing the difference in residual DNA content between the native spleen (*n* = 4) and the decellularized scaffold (*n* = 6). **(l)** Bar graph showing that there is no difference between the collagen content (hydroxyproline) between the native spleen and the scaffold (*n* = 4). **(m)** Bar graph showing that there is no difference in the content of sulfated GAGs between the native spleen and the decellularized scaffold (*n* = 4). **(n)** Bar graph showing the difference in the concentration of residual SDS content in the scaffold (*n* = 3) at two moments in the decellularization process: effluent collected at the end of the decellularization process; effluent collected after the final H_2_O wash step. All values are represented with mean ± SEM, *p < 0.05. Scale bar: 50 μm. WP, white pulp; RP, red pulp.

Macroscopic images of the spleen during decellularize processing are shown in Figure 1B. Approximately 9 h after the beginning of the process, the organ was completely translucent with an intact network of vessel-like structures remaining. Histological analysis revealing an absence of cell debris and genetic material (Figures 2a,b). These results were confirmed by the quantification of residual DNA, where a significant reduction in the amount of DNA in the decellularized scaffold was observed when compared to native tissue (4,068 ± 522 vs 51 ± 13 ng/mg dry tissue weight) (Figure 2k).

Collagen maintenance was verified through Picrosirius red staining (Figures 2c,d) and indirect quantification of the hydroxyproline amino acid (Figure 2l), and no statistical differences were observed between the native organ and the decellularized scaffold, respectively (40,038 ± 2,277 vs. 42,520 ± 6,283 μg/mg dry tissue weight). The maintenance of glycoproteins in the decellularized scaffold was demonstrated by staining with PAS (Figures 2e,f). The total content of sulfated glycosaminoglycans in the native tissue and the scaffold, respectively, was measured (Figure 2m), with no statistical difference between groups (2,216 ± 856 vs. 1,250 ± 528 μg/mg dry tissue weight).

Immunomarking showed the expression and distribution of fibronectin and laminin in the ECM of native and decellularized tissues, with maintenance of these molecules in the structure after the decellularization process (Figures 2g–j).

The residual SDS content was verified indirectly in the scaffold. There was a significant reduction in the concentration of SDS present in the effluent of the organ after washes with distilled water (0.2463 ± 0.02252% vs. 0.0120 ± 0.00057%) (Figure 2n).

**FIGURE 3:**
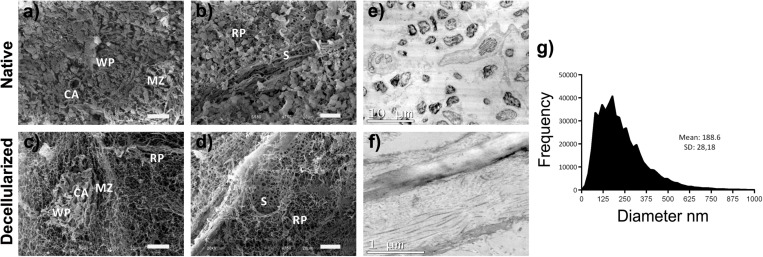
Ultrastructural analysis of the splenic scaffold. **(a–d)** Representative scanning electron micrographs showing the removal of all cells and their debris with the maintenance of the main components of the splenic parenchyma as in native tissue after decellularization of the scaffold. **(e,f)** Representative images of ultrastructural analysis by TEM demonstrating the absence of cells in the scaffold, unlike the native organ, in addition to the maintenance of the main components of the ECM, mainly collagen fibers. **(g)** Quantitative analysis of the fibers in the matrix of decellularized scaffolds showing their diversity of diameters (*n* = 3). Scale bars: **(a–d)**: 20 μm; **(e)**: 10 μm; **(f)**: 1 μm. WP, white pulp; RP, red pulp; marginal zone; CA, central artery of the white pulp; S, red pulp sinusoids.

Ultrastructural analyses showed absence of cells and cellular debris, with good preservation and organization of important structures of the splenic parenchyma, such as white pulp, marginal zone and red pulp (Figures 3a–d). Also, good preservation and integrity of the entire splenic vascular structure were observed, such as the central artery of the white pulp (CA) (Figures 3a,c), the large-caliber vessels and the red-pulp sinusoid (S) (Figures 3b,d). In the vessels, it was possible to observe the total removal of the endothelial cells after decellularization, with the maintenance of the basement membrane and structure of the vessel, as in the native organ.

The fiber diameter analysis showed variable diameters, ranging from exceedingly small fibers (20 nm thick) to large fibers (≈1,000 nm thick), with an average of 188 ± 28 nm (Figure 3g). TEM analysis reveals the ultrastructure of the different ECM components of the scaffold that are not visible in the native spleen because of the cells present (Figures 3e,f). Also, the organization and maintenance of collagen fibrillar structures with their light and dark bands can be observed (Figure 3f).

**FIGURE 4:**
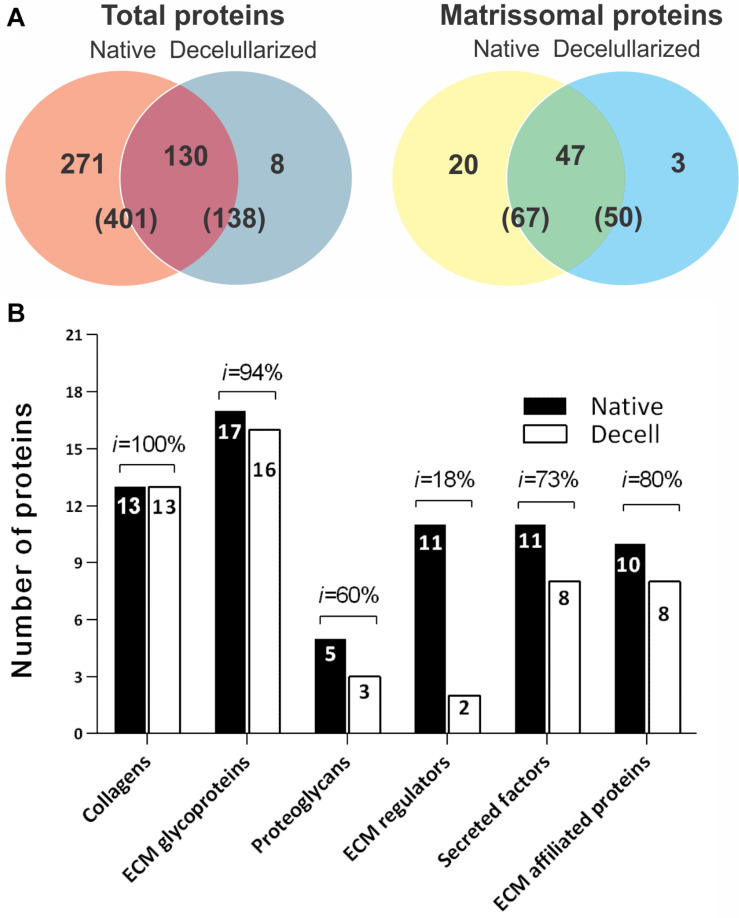
Cellular and matrisomal content before and after the spleen decellularization process. **(A)** *Venn* diagram showing the amount of total and matrix proteins shared between native and decellularized tissues. **(B)** Bar graph showing the number of proteins and the percentage maintenance rate (*i*) by class between native and decellularized tissues. The results are represented as the percentage of the total number of proteins and in parentheses, as the number of proteins for each classification.

Proteomic analysis was carried out to obtain a better understanding of the influence of the decellularization process on the splenic matrix. In total, 411 proteins were identified, of which 341 were related to cell content (Supplementary Material 1) and 70 to the splenic matrix (Table 1). Of the 411 proteins identified, 401 were present in native tissues and 138 in decellularized tissues. Regarding the constituent proteins of the matrix, 67 proteins were present in the native tissues and 50 were present in the decellularized scaffolds, with 47 shared by both tissues (Figure 4A, Table 1). Thus, there was retention of approximately 74% in the number of matrisomal protein after the decellularization process.

**TABLE 1:** List of matrix proteins present and absent in native and decellularized splenic tissues with their average intensity and percentage of retention (*n* = 4/group).

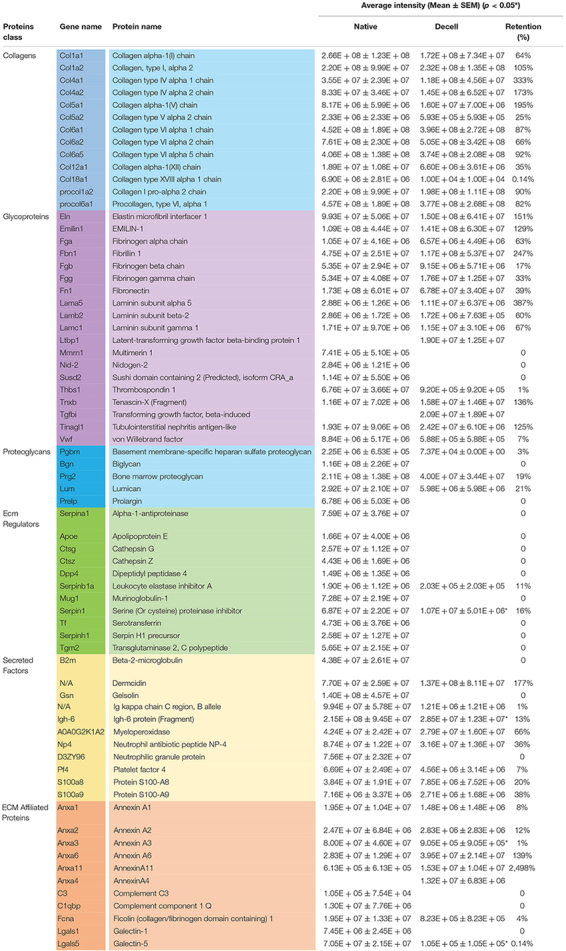

After separating the matrisomal proteins by class, it was possible to observe which ones were more or less influenced by the decellularization process. In relation to the number of proteins, the collagen subtypes obtained the highest maintenance rate (*i*) with 100% preservation, while the regulatory proteins of ECM proved to be the most sensitive to the decellularization process, with a maintenance rate of only 18% when compared to native tissue (Figure 4B).

The relationship of each matrisomal protein, and its presence or absence in native and decellularized tissues, as well as their average intensities and the percentage of intensity retention (%), are shown in Table 1. Significant differences were observed in relation to the intensity of some proteins between the native and decellularized groups, such as serine inhibitor proteinase (Serpin1); Igh-6 protein (Igh-6); Annexin A3 (Anxa3); and Galectin-5 (Lgals5). However, proteins of extreme importance for the matrix, such as the collagen and elastin subtypes, laminins and fibronectin, among others, did not obtain significant losses or even present greater intensity (Table 1).

**FIGURE 5:**
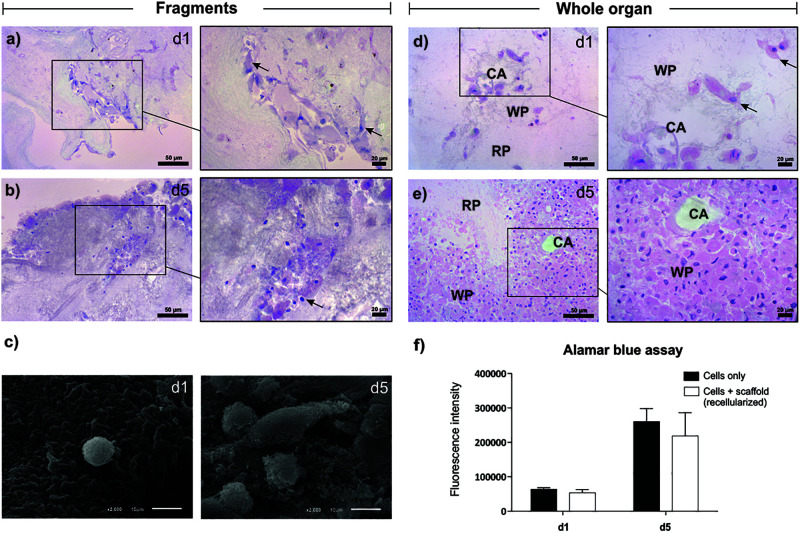
Recellularization of the splenic scaffold. Representative images of hematoxylin and eosin **(a,b)** and SEM **(c)** showing the recellularized scaffold with stromal cells (arrows) by the method of direct cultivation on the ECM (fragments), on days 1 and 5 after seeding. **(d,e)** Representative images of hematoxylin and eosin (H&E) showing the recellularized scaffold with stromal cells by the perfusion method (whole organ), on days 1 and 5 after seeding. On day 5 the cells were located mainly in the white pulp region. **(f)** Viability and proliferation assay with Alamar blue, showing the difference in fluorescence intensity produced by the metabolically active cells in the control (cells only) and recellularized (cells + scaffold) groups, after 1 and 5 days of seeding (*n* = 3/group). Abbreviations: day 1 (d1), day 5 (d5), recellularized White pulp (WP), decellularized Red pulp (RP); central artery duct of the white pulp (CA). Scale bars, H&E: 50 and 20 μm; SEM: 10 μm.

The splenic scaffolds were recellularized with stromal cells from the spleen of neonates for 5 days, using two different protocols: perfusion of cells through the spleen artery and direct cultivation over pieces of the scaffold (Figure 5). Hematoxylin and eosin staining and SEM analysis confirmed the ability of the splenic scaffold to support cell growth and adhesion. The cells increased in number from day 1 to day 5, in both seeding methods, indicating the capacity of the scaffold to support the proliferation of stromal cells (Figures 5a,b).

Through the Alamar blue assay, it was possible to confirm cell proliferation, as well as quantifying the viability of cells cultured with the scaffold. The intensity of fluorescence produced by metabolizing resazurin by viable stromal cells increased considerably from day 1 (control: 64,080 ± 4,036 vs. scaffold: 54,186 ± 8,501) to day 5 (control: 260,952 ± 37,211 vs. scaffold: 218,730 ± 67,812), both in the cultured cell group individually (control) and in the group of cells cultured in the scaffold, respectively. Also, when comparing the control and recellularized groups, there was no difference in fluorescence intensity between day 1 and day 5, demonstrating that the scaffold was not cytotoxic to cells (Figure 5c).

## Discussion

To our knowledge this is the first study in which a decellularization technique was purposely used to create an acellular and viable splenic scaffold aiming the reconstruction of the spleen. Other studies using decellularized splenic matrix as scaffold were aimed to restore liver and pancreas function (Gao et al., 2015; Xiang et al., 2015, 2016; Zheng et al., 2015; Liu et al., 2019; Vishwakarma et al., 2019).

We used a protocol adapted from Gao et al. (2015), in which rat spleens were decellularized with detergent (SDS) at a concentration lower than that used for other organs (Ott et al., 2008; Sullivan et al., 2012; Nguyen et al., 2018; Robertson et al., 2018). The lower the concentration and variety of detergents used, the greater the preservation of delicate ECM components of spleen. Also, SDS is reported to be more effective than Triton X-100 to eliminating cells in the medullary regions of dense organs and for preserving the native architecture (Nakayama et al., 2010). Unlike the study by Gao et al. (2015), we do not use the freeze/thaw method. Since freeze/thaw process can lead to a rupture of the main components of ECM, impairing the stages of recellularization and transplantation. Also, Gao et al. (2015) did not monitor the pressure during the perfusion process with detergents for organ decellularization. It can be extremely damaging to the matrix (Crapo et al., 2011). We demonstrated the maintenance of splenic parenchyma structures, such white pulp, marginal zone and red pulp, in addition to the vascular duct network (Figure 3). These is extremely important, as this will support the cells after recellularization. Also, preserving the vascular duct network is essential for the supply of nutrients and the removal of metabolites in the scaffold (Zheng et al., 2015).

We obtained an average of approximately 50 ng/mg of DNA per dry weight in the decellularized tissue. According to Crapo et al. (2011), the scaffold effectively decellularized must contain a maximum of 50 ng/mg of DNA per dry weight of ECM, as well as the absence of visible cells after staining with hematoxylin or DAPI (4′, 6-diamidino-2-phenylindole). Also, similarly to what was found in the study by Gao et al. (2015), we obtained an approximate reduction in DNA content of 99% when compared to native and decellularized tissues. We believe that the success in reducing the DNA content is partly due to the regulation of flow and pressure during decellularization process.

Retention of native ECM proteins is essential to replicate the cell niche in the decellularized ECM, as well as facilitating cell binding and signaling (Sackett et al., 2018). Collagen is the main component of ECM, and plays a key role in biomechanics, transducing tissue forces in cells (Walker et al., 2018). Collagen also modulates the phenotype and cellular functions by locally storing and releasing growth factors and cytokines during tissue repair processes (Miranda-Nieves and Chaikof, 2017). We demonstrated not only the maintenance of collagen in the decellularized scaffold but also the maintenance of its diameter. Its secondary structure was maintained, as demonstrated after the deconvolution and analysis of the spectrum of the amide I band by FTIR, in the region of 1,600–1,700 cm^–1^ (Supplementary Material 2). These results suggest the absence of a rupture of collagen structures after decellularization, as demonstrated in the study by Nara et al. (2016), which analyzed the secondary structure of collagen after corneal decellularization.

Others molecules, extremely important in the ECM, such as laminins (responsible for cell adhesion) (Nara et al., 2016), fibronectin (regulating cell adhesion, migration, and differentiation) (Mouw et al., 2014), and glycosaminoglycans were also preserved after the decellularization process. In the spleen, several matrix molecules, such as laminin, fibronectin, and collagens, constitute the basement membranes and interstitial matrices of this organ, being fundamental for the maintenance of its structure (Lokmic et al., 2008).

Proteomic analysis can confirm the previous findings, besides completely clarifying the splenic structure, through a complete analysis of the ECM of the native spleen and after decellularization. Collagen subtypes have the best retention in ECM after decellularization, with intensities very close to those of native tissues, as observed in several studies (Li et al., 2016; Sackett et al., 2018). Although some proteins are reduced in intensity in decellularized material compared to native tissue, many of them increase in intensity, which means that compared to other ECM proteins, they are more enriched after decellularization (Perea-Gil et al., 2018). Cellular proteins present in native tissues can make it difficult to identify the components of the ECM. But it is known that the use of SDS can promote enrichment of matrix proteins, facilitating the identification, by removing cellular proteins and improving the solubilization of matrix proteins (Krasny et al., 2016; Barallobre-Barreiro et al., 2017). The same is not true for proteoglycans, ECM regulators, secreted factors and proteins associated with the matrix with reduced intensity after decellularization. This was expected, since, except for proteoglycans, all other classes of molecules are not linked or are weakly linked to the ECM and are therefore easier to remove.

It is extremely important to remove the SDS residue from the scaffold, this can harm recellularization if present in high concentrations. In a study by Zvarova et al. (2016), in which the impact of residual SDS concentration in the scaffold after lung decellularization in different cell types was assessed, it was observed that there is a different SDS concentration threshold for each cell type. The residual SDS in our scaffold did not prevent the adhesion, proliferation and viability of neonatal rat splenic stromal cells when cultured with the scaffold.

After verifying the maintenance of the main components of ECM and the concentration of SDS residual, we then determined the capacity of the scaffold to support cell adhesion, growth, proliferation and survival. For that, we used splenic stromal cells from neonatal rats, as these cells (and not lymphocytes) are fundamental for the regeneration of the spleen (Tan and Watanabe, 2014). After, these authors (Tan and Watanabe, 2017), identified that the stromal cell population MAdCAM-1 + CD31 + CD201 + is essential for the formation of new splenic tissues. Despite the complete regeneration of the splenic tissue, both studies used the renal capsule to transplant stromal cells individually, either due to the large local vascularization or due to the lack of ideal support for sowing the cells. Thus, the decellularized splenic ECM functions as an ideal scaffold, serving as a support for seeding, with modulation of cellular behavior. After recellularization with patient’s own cells, the scaffold could be reimplanted in several anatomical sites, including the *peritoneum*, over the greater *omentum*, the site of anatomical origin of the spleen.

We showed that the decellularized spleen matrix can support the adhesion and proliferation of stromal cells derived from the spleen. Which reach the possibility of transplanting fragments or even the total replacement of the spleen soon. Further studies are needed to assess the ability of the splenic scaffold to support the adhesion, proliferation, and survival of other cell types, with a real possibility of application in clinical practice, such as the splenic cell pool and bone marrow cell pool. Likewise, further studies are needed to assess the *in vitro* and *in vivo* functionality of the recellularized splenic scaffold, such as in the presence of infection.

## Conclusion

We succeeded in developing a simple and efficient method for decellularization of the spleen aiming at its reconstruction. The proteome of splenic ECM was determined, and it was shown that this newly developed scaffold can support cell adhesion and proliferation and maintain the viability of splenic stromal cells after seeding. These developments can provide the basis for the reconstruction and replacement of spleen.

## Data Availability Statement

All datasets presented in this study are included in the article/Supplementary Material.

## Ethics Statement

The animal study was reviewed and approved by Institutional Ethics Committee of Universidade Federal do Espírito Santo (CEUA-UFES no. 042/2016).

## Author Contributions

TZ carried out the experimental study and acquisition, analysis and interpretation of data, and drafted the manuscript. TZ, GT, RP, and AD contributed to the adaptation and development of decellularization protocols, histology, quantification and interpretation of hydroxyproline, glycosaminoglycans, SDS, and DNA. TZ and IB participated in cell isolation, seeding scaffold, maintenance of cell culture, and data interpretation. TZ, LI, and FA participated of sample preparation method for mass spectrometry and proteomic analysis. RM participated in the supervision and in the critical revision of the manuscript. BN contributed to the conception and supervision of the study, data interpretation, and writing manuscript. All authors read and approved the final version of the manuscript.

## Conflict of Interest

The authors declare that the research was conducted in the absence of any commercial or financial relationships that could be construed as a potential conflict of interest.

## Footnotes

1 https://repository.jpostdb.org/

## Supplementary Material

Supplementary Data Sheet 1 Total proteins (cells + ECM). Total proteins of cells and extracellular matrix (ECM) identified in the proteomic analysis. All the proteomic raw data were deposited on jPOSTrepo repository (https://repository.jpostdb.org/) under the accession numbers of JPST000915 and PXD020363 for ProteomeXchange.

Supplementary Image 1 Infrared analysis of the decellularized scaffold. All spectra were observed in the range of 500 to 4,500 cm^–1^ for data acquisition, at a rate of 4 cm^–1^, with 50 scans for each sample, followed by spectral analysis for comparison. The spectrum range at 1,600–1,700 cm^–1^ was used to determine the secondary structure of proteins in native and decellularized tissues in accordance to Yang et al. (2015). **(a)** The representative comparative spectrum between the native tissue (black line) and the decellularized scaffold (gray line), showing the difference between the transmittance and the wavenumbers of each tissue. **(b)** Deconvolution representative of native and decellularized tissues (*n* = 3/group), showing the different conformations of the secondary structures of the amide I band. **(c)** Relative area (%) of each conformation calculated by the fraction corresponding to the formation of the total amide band I. The relative area of each peak was calculated using the second derivative spectrum, followed by smoothing by the Savitsky-Golay method with the different conformations determined by integration of Gaussian profiles.

Supplementary Image 2 Peroxidase *In Situ* Apoptosis Detection Assay. Typical imagens of showing the process of apoptosis in **(a)** whole organ, entire decellularized spleen on day 5th after seeding with stromal cells. Where show low nuclear staining intensity (light brown) in some cells. **(b)** Fragments of decellularized spleen on day 5th after stromal cells seeding. Also, demonstrate low nuclear staining intensity (light brown) in few cells. Positive control – ischemic kidney sections were used as a positive control, revealing many cells with nuclear marking (brown). Negative control, seeding scaffold not including anti-digoxigenin antibody (ApopTag^®^ kit, Millipore Corporation). Images at 40× objective.
