# Supplementary figures and images for: Decellularized Splenic Matrix as a Scaffold for Spleen Bioengineering
Source: Front Bioeng Biotechnol. 2020 Oct 2;8:573461. doi: 10.3389/fbioe.2020.573461 (PMC7567156; doi:10.3389/fbioe.2020.573461)

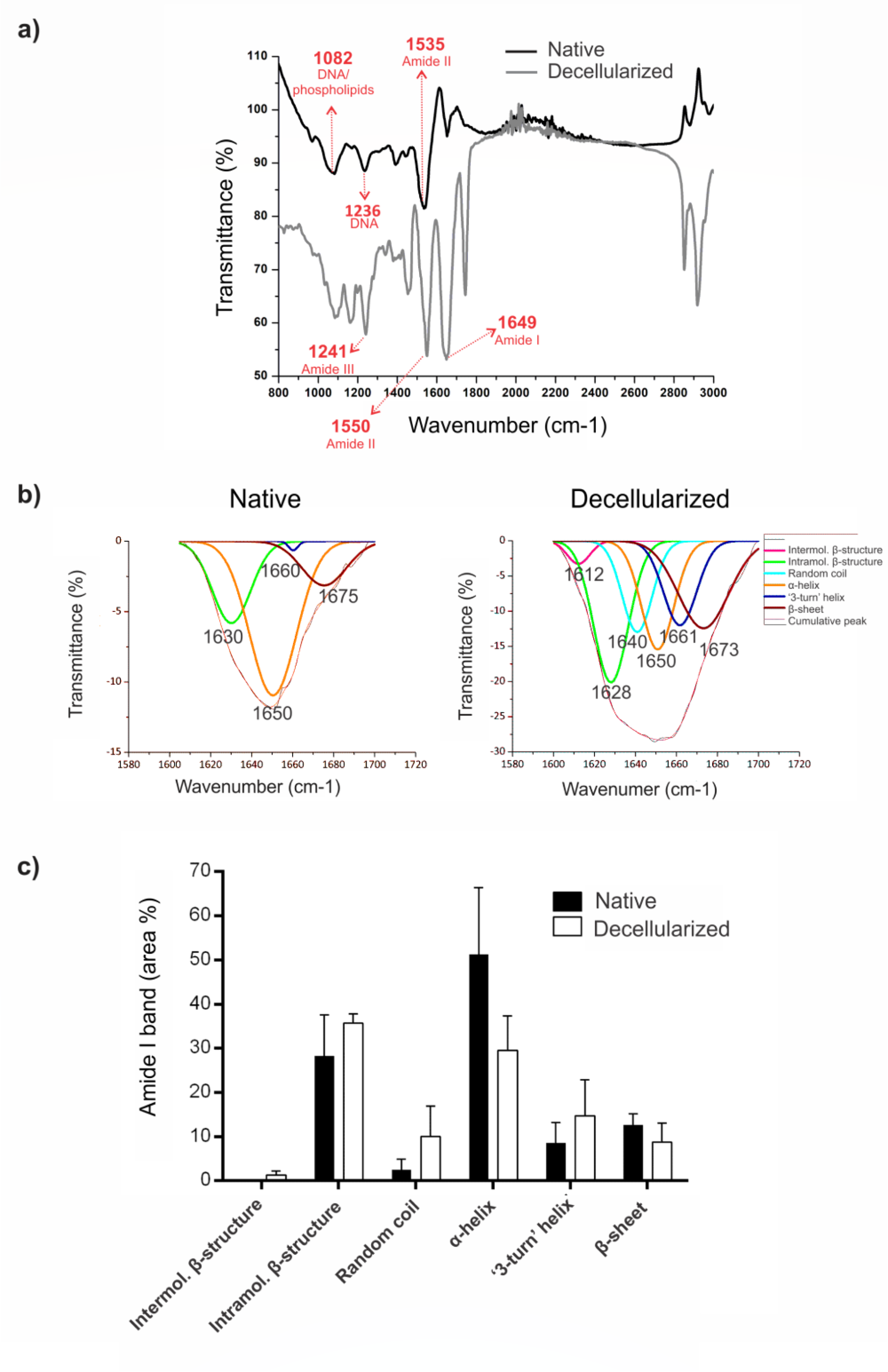

Supplement: Supplementary Image 1 — Infrared analysis of the decellularized scaffold. All spectra were observed in the range of 500 to 4,500 cm–1 for data acquisition, at a rate of 4 cm–1, with 50 scans for each sample, followed by spectral analysis for comparison. The spectrum range at 1,600–1,700 cm–1 was used to determine the secondary structure of proteins in native and decellularized tissues in accordance to Yang et al. (2015). (a) The representative comparative spectrum between the native tissue (black line) and the decellularized scaffold (gray line), showing the difference between the transmittance and the wavenumbers of each tissue. (b) Deconvolution representative of native and decellularized tissues (n = 3/group), showing the different conformations of the secondary structures of the amide I band. (c) Relative area (%) of each conformation calculated by the fraction corresponding to the formation of the total amide band I. The relative area of each peak was calculated using the second derivative spectrum, followed by smoothing by the Savitsky-Golay method with the different conformations determined by integration of Gaussian profiles. [file Image_1.tiff]

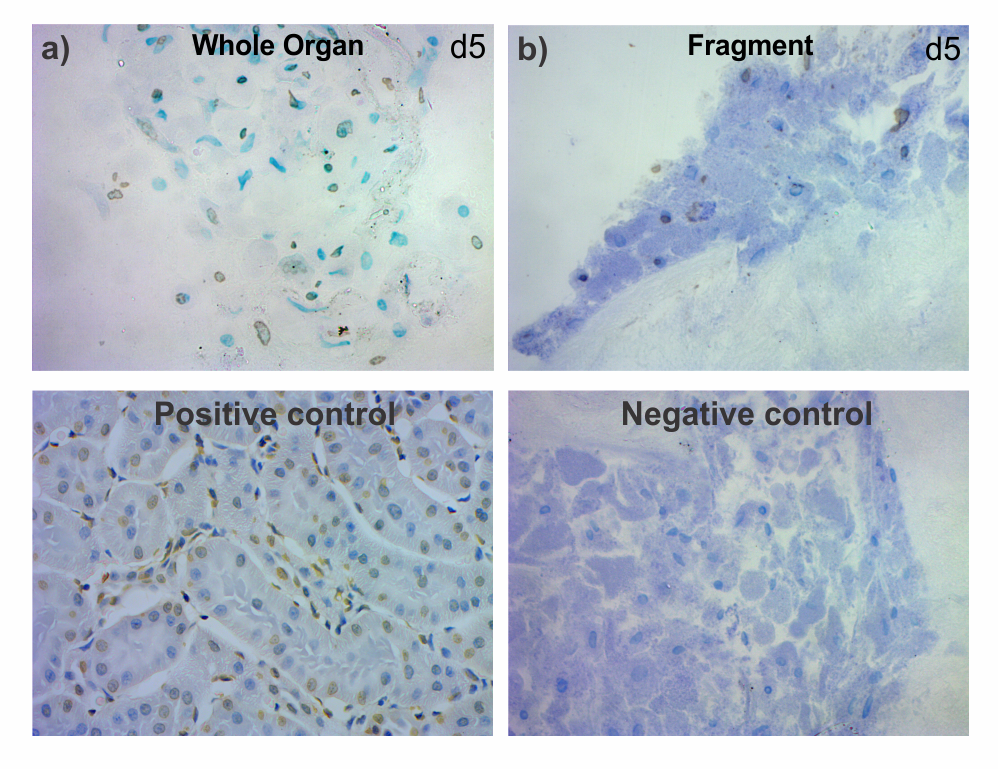

Supplement: Supplementary Image 2 — Peroxidase In Situ Apoptosis Detection Assay. Typical imagens of showing the process of apoptosis in (a) whole organ, entire decellularized spleen on day 5th after seeding with stromal cells. Where show low nuclear staining intensity (light brown) in some cells. (b) Fragments of decellularized spleen on day 5th after stromal cells seeding. Also, demonstrate low nuclear staining intensity (light brown) in few cells. Positive control – ischemic kidney sections were used as a positive control, revealing many cells with nuclear marking (brown). Negative control, seeding scaffold not including anti-digoxigenin antibody (ApopTag® kit, Millipore Corporation). Images at 40× objective. [file Image_2.TIF]
